# The association of Plk1 with the astrin–kinastrin complex promotes formation and maintenance of a metaphase plate

**DOI:** 10.1242/jcs.251025

**Published:** 2021-01-08

**Authors:** Zoë Geraghty, Christina Barnard, Pelin Uluocak, Ulrike Gruneberg

**Affiliations:** Sir William Dunn School of Pathology, University of Oxford, South Parks Road, Oxford OX1 3RE, UK

**Keywords:** Astrin, Kinastrin, SKAP, Plk1, Chromosome, Kinetochore, Mitosis

## Abstract

Errors in mitotic chromosome segregation can lead to DNA damage and aneuploidy, both hallmarks of cancer. To achieve synchronous error-free segregation, mitotic chromosomes must align at the metaphase plate with stable amphitelic attachments to microtubules emanating from opposing spindle poles. The astrin–kinastrin (astrin is also known as SPAG5 and kinastrin as SKAP) complex, also containing DYNLL1 and MYCBP, is a spindle and kinetochore protein complex with important roles in bipolar spindle formation, chromosome alignment and microtubule–kinetochore attachment. However, the molecular mechanisms by which astrin–kinastrin fulfils these diverse roles are not fully understood. Here, we characterise a direct interaction between astrin and the mitotic kinase Plk1. We identify the Plk1-binding site on astrin as well as four Plk1 phosphorylation sites on astrin. Regulation of astrin by Plk1 is dispensable for bipolar spindle formation and bulk chromosome congression, but promotes stable microtubule–kinetochore attachments and metaphase plate maintenance. It is known that Plk1 activity is required for effective microtubule–kinetochore attachment formation, and we suggest that astrin phosphorylation by Plk1 contributes to this process.

## Abstract (precis)

**Highlighted Article:** Plk1 binds to and phosphorylates the N-terminus of astrin. This promotes recruitment of the astrin complex to kinetochores and stabilises microtubule–kinetochore attachments in situations when mitosis is delayed.

## INTRODUCTION

During animal cell division the efficient end-on attachment of kinetochores to microtubules is a prerequisite for successful chromosome segregation. One of the key mitotic kinases required for the formation and maintenance of kinetochore (K-)fibres is Polo-like kinase 1 (Plk1) (Lenart et al., 2007). In mitosis, Plk1 is localised to centrosomes, centromeres and kinetochores, and has important phosphorylation targets at all of these sites (Arnaud et al., 1998; Barr et al., 2004). Localisation of Plk1 to specific loci is achieved by binding of the C-terminal polo-box-domain (PBD) to phosphorylated docking sites containing a consensus S-[pS/pT]-P/X motif (Elia et al., 2003a,b). Priming phosphorylations of these sites are often carried out by CDK1–cyclin B but can sometimes be generated by Plk1 itself (Elowe et al., 2007; Kang et al., 2006; Neef et al., 2007). Proteins of both the outer and inner kinetochore have been described as binding partners for Plk1, creating specific local pools of Plk1 activity at the kinetochore (Lera et al., 2016). How Plk1 supports the establishment of microtubule-kinetochore attachments is still not completely clear.

One described Plk1 interaction partner at the kinetochore is the mitotic spindle and kinetochore protein astrin (also known as SPAG5) (Dunsch et al., 2011; Kettenbach et al., 2018). Astrin is a long coiled-coil protein with a globular N-terminal domain, which forms a tetrameric complex with the kinetochore-associated astrin-binding partner kinastrin (also known as small kinetochore associated protein; SKAP), dynein light chain 1 (DYNLL1, also known as LC8) and the c-Myc-binding protein MYCBP (Dunsch et al., 2011; Friese et al., 2016; Gruber et al., 2002; Kern et al., 2017; Schmidt et al., 2010). Depletion of astrin or kinastrin results in severe impairment of bipolar spindle formation, failure of chromosome congression and mitotic arrest (Dunsch et al., 2011; Gruber et al., 2002; Schmidt et al., 2010; Thein et al., 2007). In particular, the formation of stable end-on microtubule–kinetochore attachments is impaired in astrin-depleted cells (Dunsch et al., 2011; Shrestha et al., 2017). The key microtubule binding protein complex at the outer kinetochore is the NDC80 complex (Cheeseman et al., 2006; Ciferri et al., 2008), and it has recently been reported that astrin helps to stabilise attachments by binding synergistically with microtubules to NDC80 (Kern et al., 2017). This process is aided by a specific pool of the PP1 phosphatase, which is delivered by astrin to kinetochores (Conti et al., 2019). Whether the astrin complex cooperates with Plk1 in stabilising microtubule–kinetochore attachments has so far not been investigated.

Here, we characterise a direct interaction between astrin and Plk1 that promotes astrin functions at the kinetochore.

## RESULTS AND DISCUSSION

### Plk1 associates with the N-terminus of astrin

**Fig. 1.**
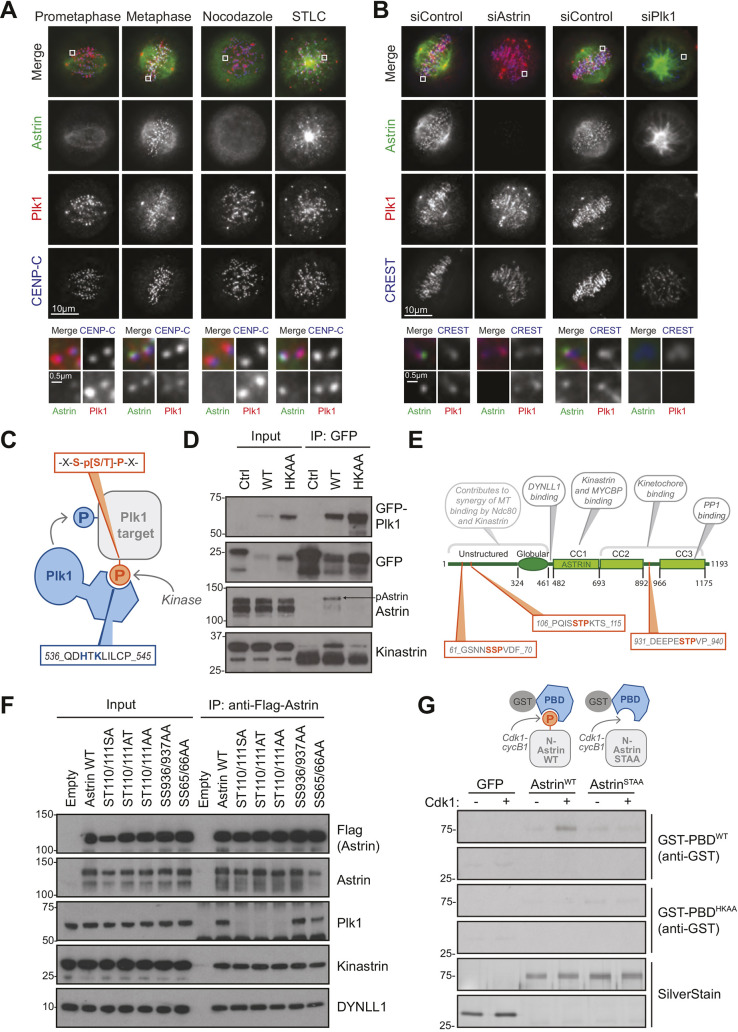
**Plk1 binds to a site in the N-terminus of astrin.** (A) Plk1 and astrin staining in HeLa cells treated as indicated. Images were scaled individually to show localisation of lower-intensity Plk1 at attached kinetochores. (B) Plk1 and astrin staining in HeLa cells depleted of astrin, Plk1 or control. Magnified images of the regions indicated are shown in the lower panels. (C) Schematic of Plk1 binding to target motifs via the PBD. (D) Plk1 WT and PBD mutants were immunoprecipitated (IP) from HEK293T cells and analysed by immunoblotting. (E) Schematic of astrin showing potential Plk1 binding sites. (F) Flag–astrin WT and S-[S/T]-P mutants were immunoprecipitated from HEK293T cells and analysed by immunoblotting. (G) GFP control or 6His–N-astrin (amino acids 1–481) were incubated with recombinant Cdk1–Cyclin B1 before far western blotting with GST–PBD^WT^ or GST–PBD^HKAA^.

Proteomic analyses of mitotic astrin or Plk1 complexes, respectively, identified Plk1 as an astrin interaction partner (Dunsch et al., 2011; Kettenbach et al., 2018), and we confirmed this by reciprocal immunoprecipitations from HeLa cells (Fig. S1A). To understand when and where during mitosis astrin and Plk1 interact, untreated HeLa cells at prometaphase or metaphase, or HeLa cells arrested in mitosis by treatment with the microtubule-depolymerising drug nocodazole or the Eg5 kinesin inhibitor S-trityl-L-cysteine (STLC) (Skoufias et al., 2006), were stained for astrin and Plk1. As reported, Plk1 associated with both centrosomes and kinetochores (Lenart et al., 2007) (Fig. 1A). Plk1 kinetochore staining was strongest on unattached kinetochores but still present at attached kinetochores at metaphase plates or in STLC-arrested cells. In contrast, astrin localised to spindle poles and attached kinetochores, as previously reported (Mack and Compton, 2001; Manning et al., 2010; Schmidt et al., 2010; Thein et al., 2007). Astrin and Plk1 thus colocalised at attached kinetochores (Fig. 1A, magnified panels at bottom). In cells depleted of astrin, Plk1 was still visible at the kinetochores of the disorganised spindles (Fig. 1B), suggesting that the bulk of kinetochore-localised Plk1 is bound by other proteins, such as BubR1 and Bub1 (Elowe et al., 2007; Jia et al., 2016; Wong and Fang, 2007). In contrast, astrin was absent from kinetochores in Plk1-depleted cells (Fig. 1B), which lack the stable kinetochore attachments required for astrin kinetochore localisation (Lenart et al., 2007; Schmidt et al., 2010), as evidenced by high levels of the spindle checkpoint protein MAD1 (also known as MAD1L1) (Fig. S1B).

Plk1 binds to phosphorylated docking sites containing the motif S-pS/pT-P/X via the PBD (Fig. 1C) (Elia et al., 2003a,b). GFP pulldowns from cells transiently expressing GFP-tagged wild-type (WT) Plk1 (GFP–Plk1^WT^) or GFP–Plk1^HKAA^, lacking two PBD residues required for phospho-specific binding (Elia et al., 2003b; Hanisch et al., 2006), confirmed that the interaction between Plk1 and astrin depended on an intact PBD, and that only the phosphorylated, high molecular mass form, of astrin interacted with Plk1 (Fig. 1D, arrow). Astrin contains three potential PBD docking sites at positions 65–67, 110–112 and 936–938 (Fig. 1E). In order to identify which of these is required for Plk1 binding, the consecutive serine and threonine residues in each motif were mutated to alanine residues. Pulldown of WT and mutated proteins from HEK293T cells followed by blotting for Plk1 revealed that mutation of S110/T111 resulted in loss of associated Plk1, whereas the equivalent double mutations at residues 936/937 and 65/66, respectively, had no effect (Fig. 1F). The docking site for Plk1 on astrin is thus composed of the sequence STP at positions 110 to 112. This site is a canonical CDK1 site and has been reported to be phosphorylated *in vivo* (Daub et al., 2008; Hegemann et al., 2011; Kettenbach et al., 2011; Nousiainen et al., 2006). To recapitulate PBD binding to astrin *in vitro*, a recombinant N-terminal region of astrin (N-astrin; amino acids 1–481) containing either the WT amino acid sequence or the S110A/T111A mutation (from here on referred to as astrin^STAA^) was phosphorylated *in vitro* with CDK1–cyclin B1 and immobilised on nitrocellulose membrane and incubated with GST-tagged PBD, before probing with anti-GST antibodies (Fig. 1G). This experiment demonstrated that PBD binding is dependent on a functional PBD (Elia et al., 2003b), as well as an intact, CDK1-phosphorylated PBD binding motif in astrin (Fig. 1C,G), consistent with the observation that the interaction between astrin and Plk1 is dependent on CDK1 activity (Kettenbach et al., 2018).

### Plk1 phosphorylates the N-terminus of astrin

**Fig. 2.**
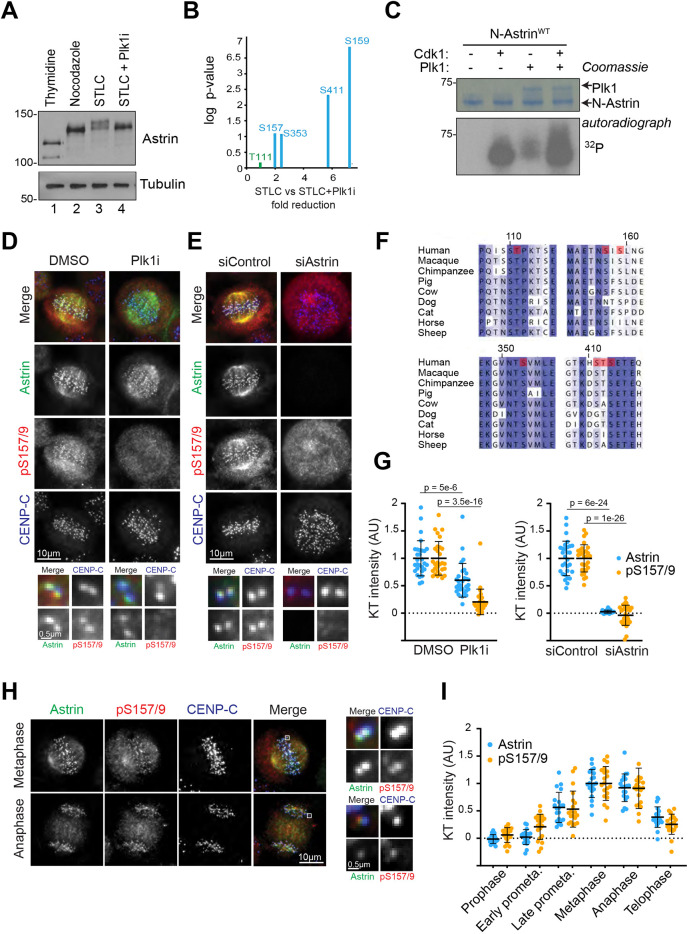
**Plk1 phosphorylates the N-terminal domain of astrin in mitosis.** (A) HeLa cells treated as indicated were lysed and analysed by immunoblotting. (B) Astrin was immunoprecipitated from HeLa cells arrested in STLC and treated for 30 mins with 1 µM Plk1 inhibitor BI2536 or DMSO control, and analysed by mass spectrometry. (C) 6His–N-astrin was incubated with recombinant CDK1–cyclin B1, recombinant Plk1 or both in the presence of [^32^P]-ATP. (D) pS157/S159 staining in HeLa cells following treatment with Plk1 inhibitor (Plk1i) or DMSO control. (E) pS157/S159 staining in control and astrin-depleted HeLa cells. Magnified images of the regions indicated are shown in the lower panels. (F) Clustal alignments of amino acid sequences surrounding the Plk1 binding and target sites in the astrin N-terminus. The phosphorylated residues are indicated in red. (G) Quantitation of kinetochore intensities for D and E, respectively. (H) pS157/S159 in asynchronous HeLa cells. Magnified images of the regions indicated are shown in the right-hand panels. (I) Quantification of the kinetochore intensities of astrin and pS157/S159. Error bars in G,I are mean±s.d. *P*-values were calculated with a two-tailed Student's *t*-test.

The docking of Plk1 to astrin suggested that the astrin complex may be a Plk1 target. This idea was corroborated by the electrophoretic running properties of astrin extracted from cells arrested in different mitotic states (Fig. 2A). Confirming previous reports, astrin was strongly upshifted, indicative of phosphorylation, in nocodazole-arrested mitotic cells (Chung et al., 2016). This upshift was further enhanced in cells arrested in STLC, mimicking conditions under which Plk1 and astrin colocalise at kinetochores (Fig. 1A). This additional upshift was reversed by incubating STLC-arrested cells with the Plk1 inhibitor BI2536 prior to cell lysis (Lenart et al., 2007) (Fig. 2A, compare lanes 2, 3 and 4), confirming that the additional upshift was due to Plk1 phosphorylation. To investigate this further, the phosphorylation status of astrin was analysed by mass spectrometry following immunoprecipitation from HeLa cells that had been arrested with STLC and mock-treated or treated with BI2536. Four sites in the astrin N-terminus (S157, S159, S353 and S411/T412/S413) (Fig. 2B, Fig. S2A) were prominent phospho-sites in the mock-treated condition but reduced in intensity by at least two-fold after BI2536 treatment (Fig. 2B, Fig. S2A) while the docking motif pThr111 was detected but unaffected by Plk1 inhibition (Fig. 2B). All four sites are conserved in most mammalian species (Fig. 2F) and fit broadly with the reported Plk1 consensus motif [E/N/D(Q)X(S/T)⌽] (Dou et al., 2011; Kettenbach et al., 2011; Nakajima et al., 2003; Santamaria et al., 2011). Interestingly, in the peptide containing S157 and S159, phosphorylation of S157 by Plk1 introduces a negative charge at the −2 position to S159 and therefore creates a sequence closer to the canonical Plk1 consensus motif for phosphorylation of S159. S411 could not be assigned with absolute certainty as the phosphorylated residue because of the presence of two S/T residues following S411 (Fig. S2A). To confirm that astrin is a substrate of Plk1, purified N-astrin was used in a radioactive kinase assay with either Cdk1–cyclin B1 or Plk1 alone, or a combination of the two kinases. While both kinases individually phosphorylated astrin, the combination of Cdk1–cyclin B1 and Plk1 resulted in the strongest [^32^P] incorporation, in keeping with the idea that Cdk1 phosphorylation of astrin promotes subsequent Plk1 phosphorylation (Fig. 2C).

Analysis of HeLa cells with an antibody raised against the doubly phosphorylated pS157/S159 peptide showed prominent staining on attached kinetochores, which was dependent on the presence of both astrin and Plk1 activity, as well as the phosphorylatable residues (Fig. 2D,E,G; Fig. S2E), and was not diminished by inhibition of other mitotic kinases (Fig. S2B,C). An antibody raised against pS353 gave a similar kinetochore staining (Fig. S2D), but also displayed spindle background staining, which was Plk1 but not astrin dependent, and this antibody was therefore not used for further analysis. Throughout mitosis, the pS157/S159 phospho-specific antibody signal largely followed the total astrin staining and, like total astrin, was retained on anaphase kinetochores, suggesting that these sites are not dephosphorylated during mitosis (Fig. 2H,I).

### Phosphorylation of astrin, but not spindle bipolarity, is dependent on the Plk1–astrin association

**Fig. 3.**
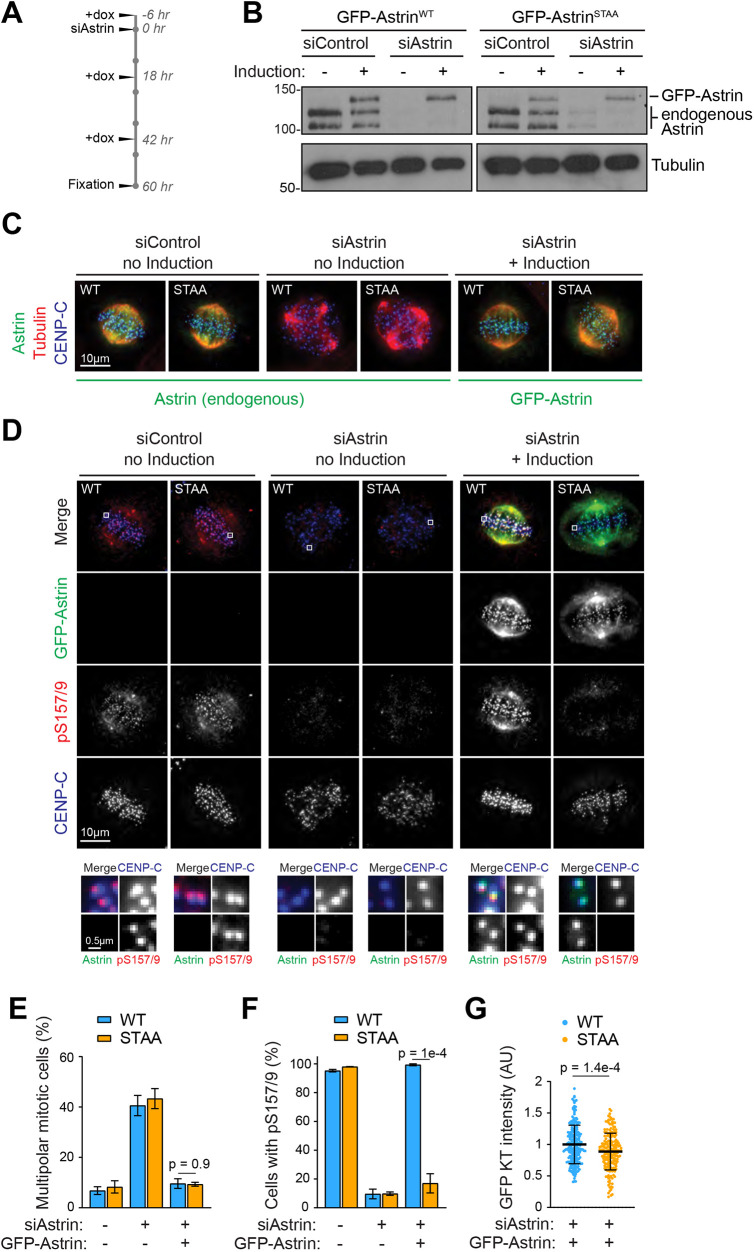
**The Plk1-binding site is required for astrin phosphorylation but not for spindle bipolarity.** (A) Timeline for astrin RNAi rescue experiments. (B) Immunoblot of HeLa Flp-In TRex cells expressing GFP–astrin^WT^ or GFP–astrin^STAA^. (C) Immunofluorescence analysis of HeLa Flp-In TRex cells expressing GFP–astrin^WT^ or GFP–astrin^STAA^. (D) Cells treated as in A were stained for pS157/S159. (E) Quantification of the percentage of multipolar mitotic cells in C. (F) Quantification of pS157/S159-positive cells in D. (G) Quantification of GFP-astrin kinetochore intensities in cells treated as in A. Error bars in E,F are s.e.m., error bars in G are s.d. All *P*-values were calculated with a two-tailed Student's *t*-test.

Depletion of astrin results in the formation of multipolar spindles, impaired chromosome congression and spindle checkpoint mediated mitotic arrest (Dunsch et al., 2011; Gruber et al., 2002; Thein et al., 2007). To test whether the association between Plk1 and astrin was relevant for the functionality of the astrin complex, RNAi rescue assays using HeLa-Flp-in cells, inducibly expressing GFP–astrin^WT^ or GFP–astrin^STAA^ at levels comparable to endogenous astrin, were carried out (Fig. 3A,B).

Replacement of endogenous astrin with GFP–astrin rescued the astrin depletion phenotype as indicated by normal progression through the cell cycle (Fig. S3A,B) and by reinstated spindle bipolarity (Fig. 3C,E). Interestingly, RNAi rescue with GFP–astrin^STAA^ restored astrin functionality to a similar extent to that seen with GFP–astrin^WT^, indicating that the association of Plk1 with the astrin complex was not necessary for the formation and maintenance of bipolar spindles, or cell cycle progression. These data are in line with the idea that the entire N-terminus of astrin may be dispensable for the function of astrin in spindle bipolarity (Kern et al., 2017). Indeed, our own analysis confirmed that GFP–astrin lacking the first 464 amino acids (GFP–astrin^ΔN^) was able to rescue spindle bipolarity and progress through an unperturbed cell cycle (Fig. S3A–D). However, Plk1-induced astrin-pS157/S159 staining was only observed in mitotic cells expressing GFP–astrin^WT^, not GFP–astrin^STAA^ (Fig. 3D–F). This suggests that Plk1 phosphorylation of astrin requires direct docking, and cannot be carried out by astrin-independent pools of Plk1 bound to other interaction partners at the kinetochore (Fig. S3E). Furthermore, we noticed that astrin kinetochore localisation was reduced in cells expressing GFP–astrin^STAA^, suggesting that the interaction of astrin with Plk1 promoted astrin kinetochore localisation (Figs 3G and 2G).

### Phosphorylation of the astrin N-terminal domain by Plk1 contributes to kinetochore–microtubule attachment stability

**Fig. 4.**
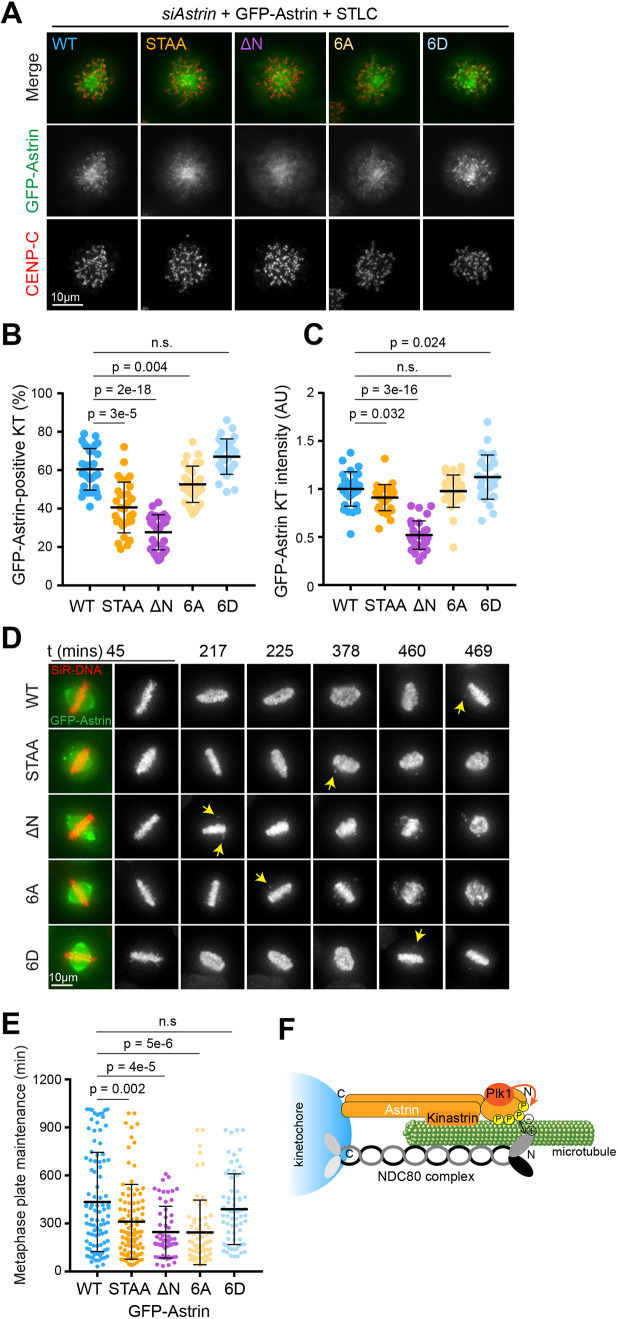
**Phosphorylation of the astrin N-terminal domain by Plk1 contributes to kinetochore–microtubule attachment stability.** (A) HeLa Flp-In TRex cells expressing GFP–astrin^WT^, GFP–astrin^STAA^, GFP–astrin^ΔN^, GFP–astrin^6A^ or GFP–astrin^6D^ were arrested overnight with STLC. (B,C) Quantification of astrin kinetochore localisation and intensity in A. (D) Live-cell imaging of MG132-arrested HeLa Flp-In TRex cells expressing GFP–astrin^WT^, GFP–astrin^STAA^, GFP–astrin^ΔN^, GFP–astrin^6A^ or GFP–astrin^6D^. Yellow arrows indicate the first chromosome loss from the metaphase plate. (E) The time from MG132 addition to first chromosome loss was plotted for cells in D. For B, C and E, each dot represents an individual cell from at least three independent repeats; error bars show mean±s.d. *P*-values were calculated with a two-tailed Student's *t*-test; ns, not significant. (F) Model illustrating how Plk1-mediated phosphorylation of the astrin N-terminus may promote binding of astrin to NDC80. For simplicity, the astrin-binding partners DYNNL1 and MYCBP are not depicted.

Our data suggested that there may be a function of astrin-associated Plk1 that was not captured by assessing spindle bipolarity or cell cycle progression under unperturbed conditions. We hypothesised that the promotion of astrin localisation to kinetochores by Plk1 may only become necessary when microtubule–kinetochore interactions are put under additional stress, for example, by a prolonged mitotic arrest. Hence, we analysed the ability of different astrin mutants to localise to kinetochores in STLC-arrested monopolar cells (Fig. 4A–C). In these cells, GFP–astrin^WT^ was localised on average to 60.5±10.8% (mean±s.d.) of kinetochores, whereas GFP–astrin^STAA^ only localised to 46.8±12.4% of kinetochores. Strikingly, GFP–astrin^ΔN^ lacking the entire N-terminus was only found at 27.7±9.1% of kinetochores and with a much-reduced intensity. Interestingly, a version of astrin in which the four Plk1 sites (S157, S159, S353, S411) as well as the two phosphorylatable serine/threonine residues following S411 (T412, S413) had been mutated to alanine (GFP–astrin^6A^), showed a similarly impaired kinetochore localisation to that of GFP–astrin^STAA^, whereas the phospho-mimetic mutant GFP-astrin^6D^ behaved like wild type (Fig. 4A–C).

To further assess how the different versions of astrin promoted microtubule–kinetochore attachments in STLC-treated cells, the analysis of GFP–astrin was combined with a 9 min cold treatment (Fig. S4A–C). Under these conditions, only stable microtubule–kinetochore attachments are preserved (Rieder, 1981). In this situation, the contribution of the astrin N-terminus to kinetochore localisation and attachment stabilisation was particularly evident, as in cold-treated STLC spindles GFP-astrin^ΔN^ was completely lost from kinetochores (Fig. S4A–C), whereas GFP–astrin^WT^ and, with lower efficiency, GFP–astrin^STAA^, still localised to kinetochores. Again, GFP–astrin^6A^ and GFP–astrin^6D^ behaved similarly to the STAA and WT variants, respectively. Measurements of the total cold-stable microtubule intensity in these cells confirmed that cells with less kinetochore-localised astrin also had fewer cold-stable microtubules (Fig. S4D,E), supporting a direct relationship between astrin kinetochore localisation and the stabilisation of kinetochore-associated microtubules.

To further test whether the astrin–Plk1 complex was required for the maintenance of stable microtubule–kinetochore attachments, mitotic cells expressing the different GFP–astrin variants were treated with the proteasome inhibitor MG132 to prevent anaphase onset, and assessed for their ability to maintain a metaphase plate (Fig. 4D,E). Cells expressing GFP–astrin^WT^ upheld a metaphase plate on average for 434±310 min (mean±s.d.) after MG132 addition before chromosomes started to leave the plate. This time was reduced to 311±234 min in the GFP–astrin^STAA^ mutant and even further to 246±161 min in GFP–astrin^ΔN^. GFP–astrin^6A^ also showed reduced plate stability, whereas GFP–astrin^6D^ behaved like GFP–astrin^WT^. Taken together with the localisation data in Fig. 4B, these data suggest that the presence of the Plk1-phosphorylated astrin N-terminus promotes the accumulation of the astrin complex at attached kinetochores, without which attachments appear more prone to dissociate. This becomes particularly important when microtubule–kinetochore attachments have to be maintained for longer periods of time.

### Astrin and Plk1 promote microtubule–kinetochore attachments synergistically

Both the astrin complex and the Plk1 kinase have been implicated in promoting the formation of stable microtubule–kinetochore attachments and here we have investigated how these two factors synergise. It has previously been shown that the C-terminus of astrin contains the main kinetochore-binding site and that the N-terminus alone does not localise to any spindle structures (Conti et al., 2019; Dunsch et al., 2011; Kern et al., 2017). Our data here suggest that the Plk1-phosphorylated astrin N-terminus facilitates the recruitment of the astrin complex to kinetochores and thus promotes the formation of stable microtubule–kinetochore attachments. Localisation of the astrin complex to kinetochores is negatively regulated by Aurora B (Manning et al., 2010; Schmidt et al., 2010), and we speculate that an interaction between unphosphorylated (positively charged) Aurora B sites in the N-terminus of NDC80, and the phosphorylated (negatively charged) astrin N-terminus, may support the recruitment of the astrin complex to bioriented kinetochores (Fig. 4F).

## MATERIALS AND METHODS

### Chemicals and antibodies

General laboratory chemicals and reagents were obtained from Sigma-Aldrich and Thermo Fisher Scientific. Drugs were dissolved in DMSO unless specifically indicated. Inhibitors were obtained from Sigma-Aldrich (Eg5 inhibitor STLC, 10 mM stock), Selleck (Plk1 inhibitor BI2536, 2 mM stock), Millipore (Aurora B inhibitor ZM447439, 10 mM stock), Cambridge Bioscience (Aurora A inhibitor MLN8237, 10 mM stock), Tocris (MPS1 inhibitor AZ3146, 20 mM stock; PP1/PP2A inhibitor Calyculin A, 1 mM stock), Insight Bioscience (proteasome inhibitor MG132, 20 mM stock) and Merck (microtubule polymerisation inhibitor nocodazole, 0.66 mM stock). Thymidine (Sigma-Aldrich, 100 mM stock) and doxycycline (Invivogen, 2 mM stock) were dissolved in water. Recombinant CDK1–cyclin B1 and Plk1 were purchased from Thermo Fisher Scientific (PV3292) and Promega (V2841), respectively. ATP-γ-[^32^P] was obtained from Perkin Elmer (BLU502A250UC). DNA vital dye SiR-Hoechst (Spirochrome) was dissolved in DMSO and used at 100 nM final concentration.

Commercially available polyclonal (pAb) or monoclonal (mAb) antibodies were used for tubulin [mouse mAb; Sigma, (DM1A) T6199, 1:2000 dilution], Plk1 (mouse mAb; Santa Cruz Biotechnology, clone F-8, sc-17783, 1:1000 dilution), Mad1 (rabbit pAb; GeneTex GTX105079, 1:2000 dilution), CENP-C (guinea pig pAb; MBL, PD030, 1: 2000 dilution). Human CREST serum was obtained from Antibodies Inc (15-234-0001, 1:1000 dilution). Antibodies against astrin and kinastrin have been described before (Dunsch et al., 2011; Thein et al., 2007). Antibodies against the doubly phosphorylated astrin pS157/S159 peptide CMAETN(pS)I(pS)LNGP and pS353 peptide CKGVNTSVMLEN, coupled to KLH, were raised in sheep (Orygen Antibodies). The antibodies were affinity-purified from the serum on the immobilised immunising peptide and used at 0.1 µg/ml. Sheep-anti-GFP antibody was a gift from Francis Barr (University of Oxford, UK).

Secondary donkey antibodies against mouse, rabbit, guinea pig or sheep IgG and labelled with Alexa Fluor 488, Alexa Fluor 555, Alexa Fluor 647, Cy5 or HRP were purchased from Molecular Probes and Jackson ImmunoResearch Laboratories, Inc., respectively. Affinity purified primary and HRP-coupled secondary antibodies were used at 1 µg/ml final concentration. For western blotting, proteins were separated by SDS-PAGE and transferred to nitrocellulose using a Trans-blot Turbo system (Bio-Rad). Protein concentrations were measured by Bradford assay using Protein Assay Dye Reagent Concentrate (Bio-Rad). All western blots were revealed using ECL (GE Healthcare).

### Molecular biology and siRNA reagents

Astrin mutant expression constructs were made using pcDNA5/FRT/TO vectors (Invitrogen) modified to encode the EGFP or FLAG reading frames. Mutagenesis to introduce phospho-site mutations and resistance to astrin siRNA was performed using the QuikChange method (Agilent Technologies). DNA primers were obtained from Invitrogen. For the knockdown of Plk1 siRNA (siPlk1) duplexes of 5′-AACGAGCTGCTTAATGACGAGTT-3′ were used (Thermo Fisher Scientific). For astrin siRNA (siAstrin) oligo #367 (5′-TCCCGACAACTCACAGAGAAA-3′) (Qiagen) was used as described previously (Thein et al., 2007). As a control siRNA, 5′-CGTACGCGGAATACTTCGA-3′ (siGl2, targeting firefly luciferase) was used.

### Cell culture and transfection

HeLa cells (ATCC) and HEK293T cells (ATCC) were cultured in DMEM with 1% (v/v) GlutaMAX (Life Technologies) and 10% (v/v) bovine calf serum at 37°C and 5% CO_2_. HeLa Flp-In TRex cells expressing GFP–astrin variants were maintained in medium supplemented with 200 μg/ml hygromycin B (Invivogen) and 4 μg/ml blasticidin (Invivogen). For plasmid transfection and siRNA transfection, Mirus LT1 (Mirus Bio LLC) and Oligofectamine (Invitrogen), respectively, were used. HeLa cell lines with single integrated copies of the desired transgene were created using the T-Rex doxycycline-inducible Flp-In system (Invitrogen) (Tighe et al., 2004); HeLa-Flp-In TRex parental cells were a generous gift from Stephen Taylor (University of Manchester, UK). All cell lines were regularly checked for mycoplasma contamination.

### Immunofluorescence analysis

Cells were fixed with PTEMF fixation buffer (20 mM Pipes-KOH, pH 6.8, 0.2% Triton X-100, 1 mM MgCl2, 10 mM EGTA and 4% formaldehyde) for 12 mins at room temperature (RT) (Dunsch et al., 2011). Coverslips were washed three times with PBS before transfer into blocking solution (PBS with 3% BSA) and blocking proceeded for 30 mins. For immunofluorescence with phospho-specific antibodies (Fig. 2; Figs S2, S3), calyculin A was added to fixative at 0.1 µM and to blocking solution at 0.025 µM. Primary and secondary antibody incubations were performed in blocking solution for 1 h and 30 min, respectively. DNA was stained in the secondary antibody incubation with 1 μg/ml DAPI. Coverslips were washed three times with PBS after each incubation. Coverslips were dried and mounted in moviol on microscope slides.

For Fig. S4, cells on coverslips were subjected to 9 min cold treatment [incubation in 4°C DMEM plus 10% FBS (w/v) in an ice/water bath] prior to fixation with PTEMF pre-warmed to 37°C for 15 min, allowing the loss of depolymerized tubulin from the cytoplasm and the exclusive preservation of cold-stable microtubules.

### RNAi rescue experiments

For astrin siRNA rescue experiments, HeLa Flp-In TRex cells inducibly expressing GFP–astrin^WT^ or mutants thereof were used. Astrin siRNA rescue was performed by induction with 2 µM doxycycline of GFP–astrin transgenes resistant to astrin RNAi, for 6 h prior to a 60 h siRNA depletion of endogenous astrin using oligonucleotide #367. A second induction was performed 18 h into the siRNA depletion. For live-cell imaging, cells were treated with 2 mM thymidine 18 h after RNAi addition, for 18 h. The thymidine was removed by washing three times with DMEM, with 2 µM doxycycline re-added in the final wash. SiR-DNA (Spirochrome) was added to the final wash of the thymidine release at a concentration of 100 nM, and imaging commenced 9 h later. Live-cell imaging was performed on a Deltavision Elite system using an inverted microscope (IX81; Olympus) and equipped with a QuantEM EMCCD camera (Photometrics). Cell were placed in a 37°C and 5% CO_2_ environmental chamber (Tokai Hit) on the microscope stage with lens heating collar. Imaging was performed using a 60× NA 1.4 oil immersion objective lens. Cells were imaged using 5% 488 nm laser power with 50 ms exposure for GFP–astrin and 2% 647 nm laser power with 10 ms exposure for SiR-DNA. A total of 7 axial planes were captured at 2 μm apart (*Z* then wavelength) at an interval of 2 min for 12 h to analyse mitotic progression. For live-cell imaging of MG132-arrested cells, 9 h after thymidine release MG132 was added to a final concentration of 20 μM immediately before cells were placed on the microscope, and image capture was begun as soon as possible. A total of 11 axial planes were captured at 2 μm apart (*Z* then wavelength) at an interval of 3 min for 16 h. These images were then used to determine when the first chromosomes left the mitotic metaphase plate. For the majority of analysed cells, which were already at metaphase when imaging commenced, the set up time for that experiment (from MG132 addition to start of imaging) was added during analysis; for a minority of cells, which entered mitosis during imaging, the time from last chromosome congression to first chromosome loss was measured.

### Purification of recombinant astrin fragments

N-astrin^WT^ and N-astrin^STAA^ (amino acids 1–481) were cloned into pQE32 and purified from *E. coli* JM109 cells on Ni-NTA agarose (Qiagen) following standard procedures.

### Radioactive kinase assay

For kinase assays, 1 µg recombinant His–N-astrin (amino acids 1–481) was incubated with control (buffer) or 100 ng recombinant Cdk1–cyclin B1 (ThermoFisher), Plk1 (Promega) or both kinases (100 ng each), for 20 min at 30°C in 50 mM Tris-HCl, pH 7.3, 50 mM KCl, 10 mM MgCl2, 20 mM sodium β-glycerophosphate, 15 mM EGTA, 0.1 mM ATP, 1 mM DTT, and 2 µCi (0.04 MBq) [^32^P]γ-ATP per reaction, in a total reaction volume of 20 µl. The reactions were stopped by the addition of 5× Laemmli buffer and boiling.

### Far western blot analysis

1 µg of purified His-tagged N-terminal astrin (amino acids 1–481) containing WT or S110A/T111A mutations, respectively, was incubated with 10 μl 2× MEB (100 mM Tris-HCl pH7.4, 100 mM KCl, 20 mM MgCl2, 40 mM sodium β-glycerolphosphate, 30 mM EGTA), 1 μl of 10× ATP/DTT mix (10 mM ATP, 100 mM DTT), 100 ng Cdk1–cyclin B1 (Thermo Fisher Scientific) and made up to 20 μl in water. Reactions were incubated for 60 mins at 30°C. Samples were denatured in Laemmli buffer, run on 10% SDS-PAGE gels and transferred onto nitrocellulose membrane. The membrane was incubated overnight in blocking buffer (50 mM Tris-HCl pH 7.5, 137 mM NaCl, 0.1% Tween 20 and 4% milk powder). Samples were incubated with 1 μg/ml His-GST-tagged Plk1-PBD constructs (either WT or HKAA mutants) for 8 h at RT. The membrane was then washed 3x in blocking buffer and incubated overnight with rabbit anti-GST antibody before three washes (PBS plus 0.1% Tween 20) before incubation with donkey-anti-rabbit IgG secondary antibody coupled to HRP.

### FLAG pulldown assays

Flag-tagged astrin constructs were transiently transfected into HEK293T cells using TransIT-LT1 transfection reagent (Mirus Bio) according to the manufacturer's protocol. At 24 h post transfection, cells were arrested with 10 µM STLC for 14 h overnight. Cells were collected via a mitotic shake-off and lysed in 50 mM Tris-HCl, pH 8.0, 150 mM NCl, 1% IGEPAL, protease inhibitor cocktail (Sigma) and phosphatase inhibitor cocktail 3 (Sigma) prior to immunoprecipitation with FLAG–agarose beads (Sigma).

### Immunoprecipitations

For the analysis of astrin phosphorylation sites, HeLa cells stably expressing GFP–astrin (Dunsch et al., 2011) were arrested overnight in STLC. One half of the cells was treated for 30 mins with 1 µM Plk1 inhibitor BI2536 prior to harvest by mitotic shake-off; the other half was left untreated. Cell pellets were lysed [50 mM HEPES pH8, 100 mM KCl, 1%IGEPAL, 0.25% Triton X-100, 1 mM DTT, 1:250 protease inhibitor cocktail (Sigma), 1:100 phosphatase inhibitor cocktail 3 (Sigma), 50 mM EDTA, 1 mM PMSF and 50 U micrococcal nuclease] for 30 mins at 4°C before centrifugation (20,800 ***g*** for 15 min). Per 1 mg of cell lysate 1 μg of sheep anti-GFP antibody (a kind gift of Francis Barr, University of Oxford, UK) was added. Immunoprecipitated proteins were eluted first with 0.1 M glycine, pH 2.6 followed by 50 mM Tris-HCl, pH 8.5, and 8 M urea. Western blots were used to confirm the success of the immunoprecipitations.

### Mass spectrometry

For mass spectrometry analysis, samples were processed using filter-aided sample preparation (FASP) columns. Samples were reduced using 10 mM tris(2-carboxyethyl)phosphine hydrochloride (TCEP) for 30 min followed by alkylation, using 20 mM chloroacetaldehyde (CAA), in the dark for 30 min. Proteins were digested with 1.5 μg trypsin (Promega) at 37°C for 12 h. Samples were reduced to ∼50 μl using a Thermo Scientific SpeedVac concentrator centrifuge.

Phospho-peptide enrichment was performed using titanium dioxide microspin columns (TopTip; Glygen). All spin steps were performed at 550 rpm, equivalent to 34 ***g***, for 5 min at room temperature. Columns were stripped with 50 μl elution buffer (5% ammonia solution in water) and washed three times with 65 μl loading buffer [1 M glycolic acid, 80% acetonitrile (ACN), 5% trifluoroacetic acid (TFA)]. Samples were diluted 1:1 using concentrated loading buffer (10% TFA, 2 M Glycolic acid, 80% ACN) and loaded onto the column 65 μl at a time. Following loading, columns were washed once each with loading buffer, wash buffer (0.2% TFA acid in 80% ACN) and finally 20% ACN. Phospho-peptides were eluted using 2×10 μl of elution buffer into an Eppendorf containing 20 μl 5% formic acid. Liquid chromatography was performed using an EASY-nano-LC 1000 system (Proxeon). Peptides were loaded onto a 75 μm internal diameter guard column (packed with Reprosil-Gold 120 C′8, 3 μm, 120 Å pores) using Solvent A [0.1% (v/v) formic acid in water] at 500 bar. Peptide separation was performed by an EASY-Spray column at 45°C (50 cm×75 µm ID, PepMap RSLC C18, 2 µm; Thermo Fisher Scientific). A 30 min linear 8–30% (v/v) ACN gradient was used with a flow rate of 200 nl/min. An EASY-Spray nano-electrospray ion source was used to introduce peptides into a Q-Exactive mass spectrometer and spectra were acquired with an *m*/*z* range of 350–1500. The 20 most abundant peaks were fragmented using CID. MaxQuant (Tyanova et al., 2016a) was used to process the data, and Perseus was used to analyse the mass spectrometry data sets (Tyanova et al., 2016b). Full MS data are available upon request.

### Quantification and statistical analysis

Image analysis was performed in FIJI and Excel (Microsoft). Eight *Z*-stacks with a 0.2 µm interval were maximum projected for analysis. Relative protein kinetochore intensities were determined by placing a 5-pixel-wide circular region of interest over individual kinetochores and measuring the mean pixel fluorescence. For immunofluorescence analysis, but not when measuring GFP-tagged proteins, this was then divided by the mean pixel intensity of the CENP-C channel within the same region of interest (ROI); and a mean background (cytoplasm) intensity for each cell was subtracted from each kinetochore protein or CENP-C measurement.

To ensure that the analysis of GFP–astrin-expressing HeLa Flp-in cells (in Figs 4 and 3G; Fig. S4A–C) was not affected by varying GFP–astrin expression levels potentially leading to different GFP–astrin kinetochore levels, these cells were fixed in PFA to preserve the cytoplasmic pool of astrin, the intensities of the whole cells were measured, and only cells with equivalent expression levels were included in the analysis. For the analysis of GFP–astrin kinetochore intensities in Figs 3 and 4 and Fig. S4, for each kinetochore, a ‘donut’-shaped ROI of background intensity was measured around the kinetochore ROI and the individual background intensity subtracted. For all fluorescence intensity analysis, the mean protein fluorescence of each kinetochore was divided by the mean kinetochore intensity of the total control population to generate relative values, which were plotted as bar graphs or scatter plots. In scatter plots, each dot represents the average intensity of 20 kinetochores from a single cell, except in Fig. 3G and Fig. S2C, in which each dot represents a kinetochore. A total of 10 cells were quantified per condition in three independent repeats. Unless otherwise stated, for kinetochore quantifications, averages are indicated by the bars and error bars represent the s.d. For the quantification of multipolar cells in Fig. 3E and the quantification of phospho-astrin staining in Fig. 3F, bars represent the mean±s.e.m. of four independent experiments, with 50–150 cells counted per condition per repeat. For Fig. 4B, the number of astrin-positive kinetochores was counted and calculated as a percentage of visible kinetochores from CENP-C staining. For each condition 30 cells were analysed from two or three independent repeats. In the scatter plot each dot represents a single cell; the error bars show mean±s.d. For the quantification of kinetochore intensities in Fig. 4C, each dot represents the mean kinetochore intensity of an individual cell and the error bars shown are mean±s.d. For Fig. S4D, the GFP–astrin signal was used to determine the outline of STLC-arrested mitotic cells, which were then measured for tubulin intensity and corrected to local background; in the graphs each dot represents the total tubulin intensity of an individual cell and the error bars shown are mean±s.d.

Production of graphs was performed on GraphPad Prism (GraphPad Software, Inc.) using data exported from Excel. Statistical analysis of kinetochore intensities was carried out in Excel or GraphPad Prism. All *P*-values were calculated by two-tailed Student *t*-test.

## Supplementary Material

Supplementary information

Reviewer comments
