# Supplementary material for: The association of Plk1 with the astrin–kinastrin complex promotes formation and maintenance of a metaphase plate
Source: J Cell Sci. 2021 Jan 8;134(1):jcs251025. doi: 10.1242/jcs.251025 (PMC7803464; doi:10.1242/jcs.251025)
Supplement: Supplementary information [file joces-134-251025-s1.pdf]

Figure S1

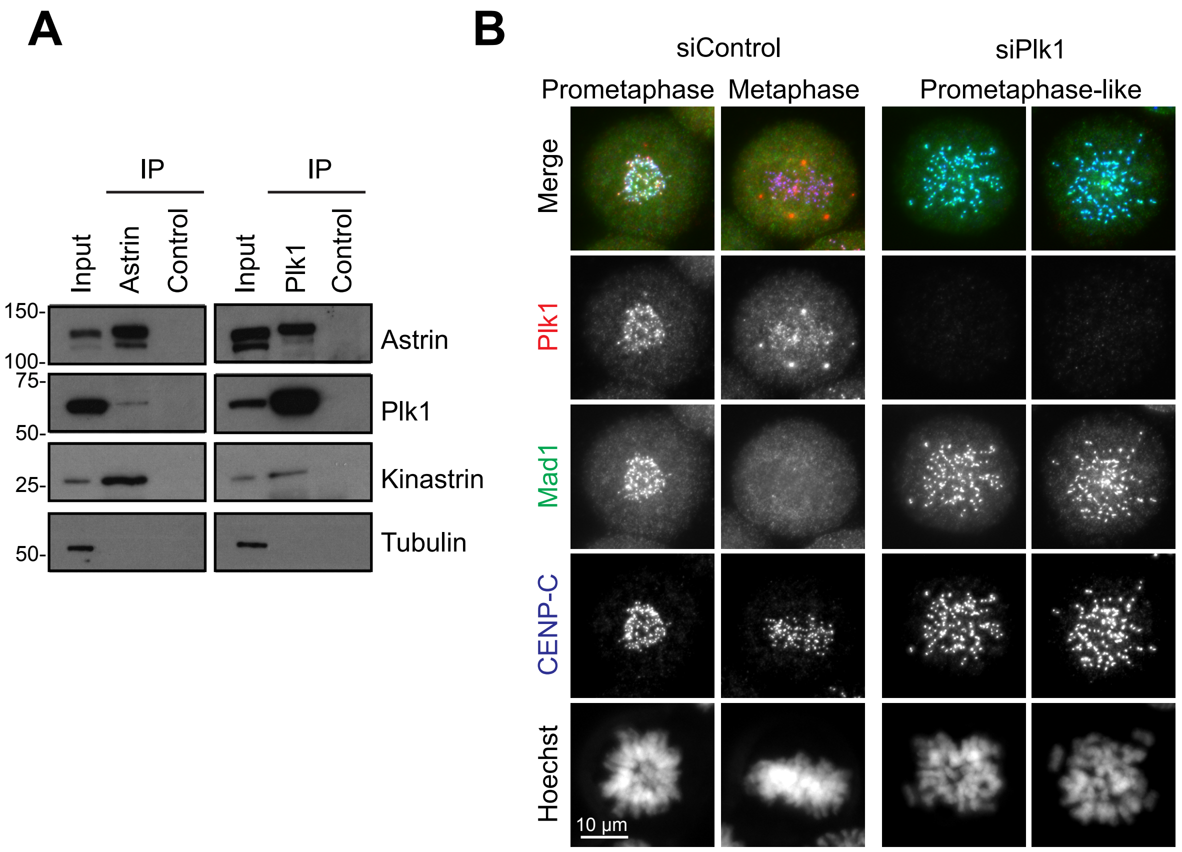

Figure S1. Astrin and Plk1 interact biochemically. **A)** Immunoprecipitations of Astrin (left) and Plk1 (right) from STLC-arrested HeLa cells were analysed by Western blotting with the indicated antibodies. **B)** Control HeLa cells or HeLa cells depleted of Plk1 were stained with antibodies against Plk1, Mad1 and CENP-C. DNA was visualised with Hoechst DNA dye.

Journal of Cell Science • Supplementary information

Figure S2

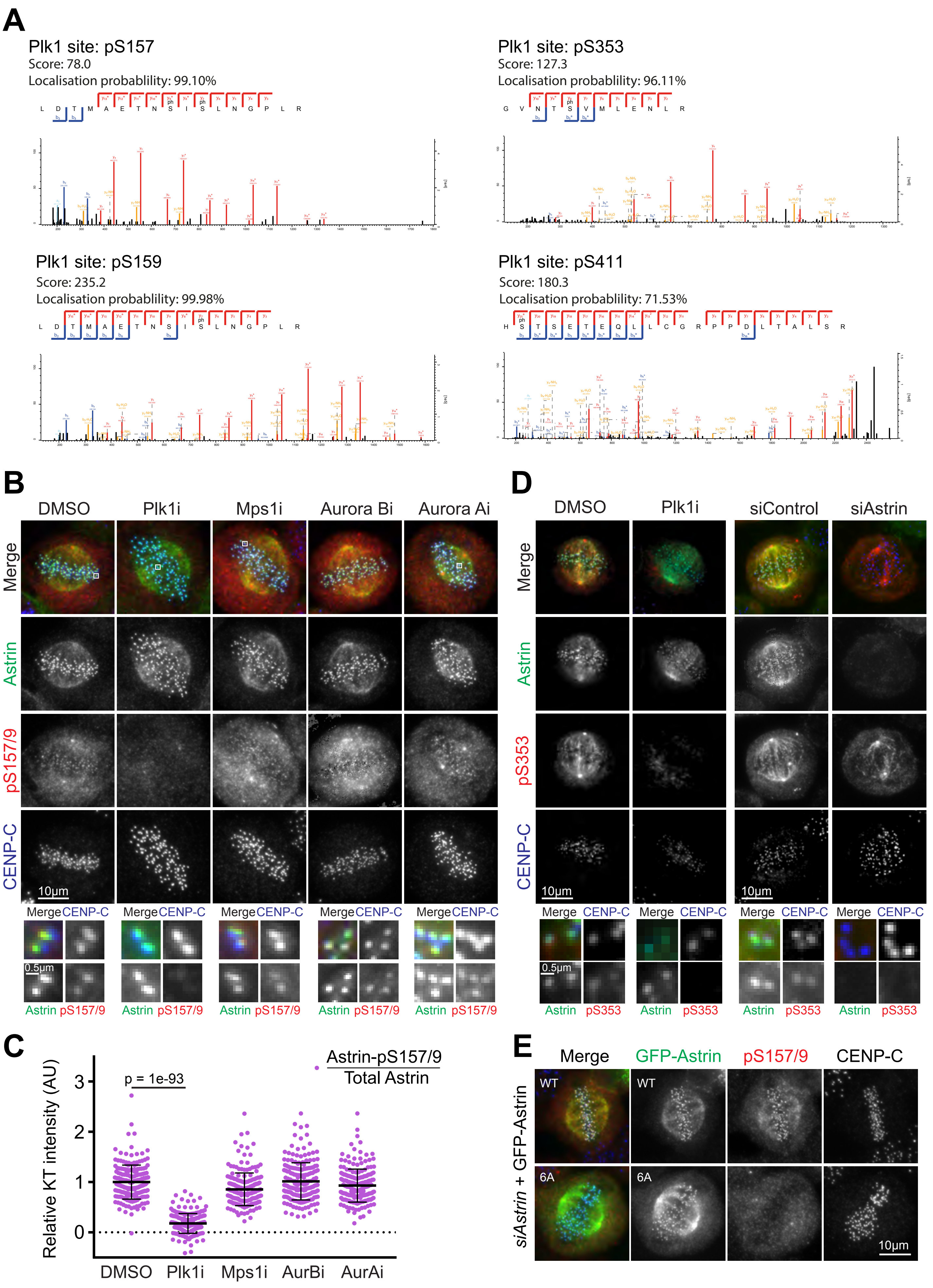

Journal of Cell Science • Supplementary information

Figure S2. Plk1 phosphorylates four sites in the N-terminus of Astrin. **A)** Four Plk1 phosphorylation sites in Astrin were identified by mass spectrometry. **B)** HeLa cells were incubated for 15 mins with inhibitors specific for Plk1 (BI2536, 1μM), Mps1 (AZ3146, 2 μM), Aurora B (ZM447439, 2 μM) and Aurora A (MLN8237, 0.5 μM), or DMSO as a control. The cells were then fixed and stained with antibodies against total and pS157/9 Astrin. **C)** Kinetochore intensities for pS157/9 and Astrin were measured for cells shown in B, and normalised to CENP-C intensity. These measurements are plotted as the ratio of pS157/9 intensity relative to total Astrin intensity. Each dot represents an individual kinetochore; bars represent the mean ±SD. **D)** Astrin pS353 was analysed by immunofluorescence staining with a pS353 antibody in HeLa cells. Left panel shows staining following 30min treatment with Plk1 inhibitor or DMSO control; right panel shows staining in control- and Astrin-depleted cells. **E)** HeLa Flp-In TRex cells depleted of endogenous Astrin and induced to express GFP-Astrin^{WT} or GFP-Astrin^{6A} (S157, S159, S353 and STS411-3 mutated to alanine). The cells were then fixed and stained with the phospho-specific pS157/9 Astrin antibody.

Journal of Cell Science • Supplementary information

Figure S3

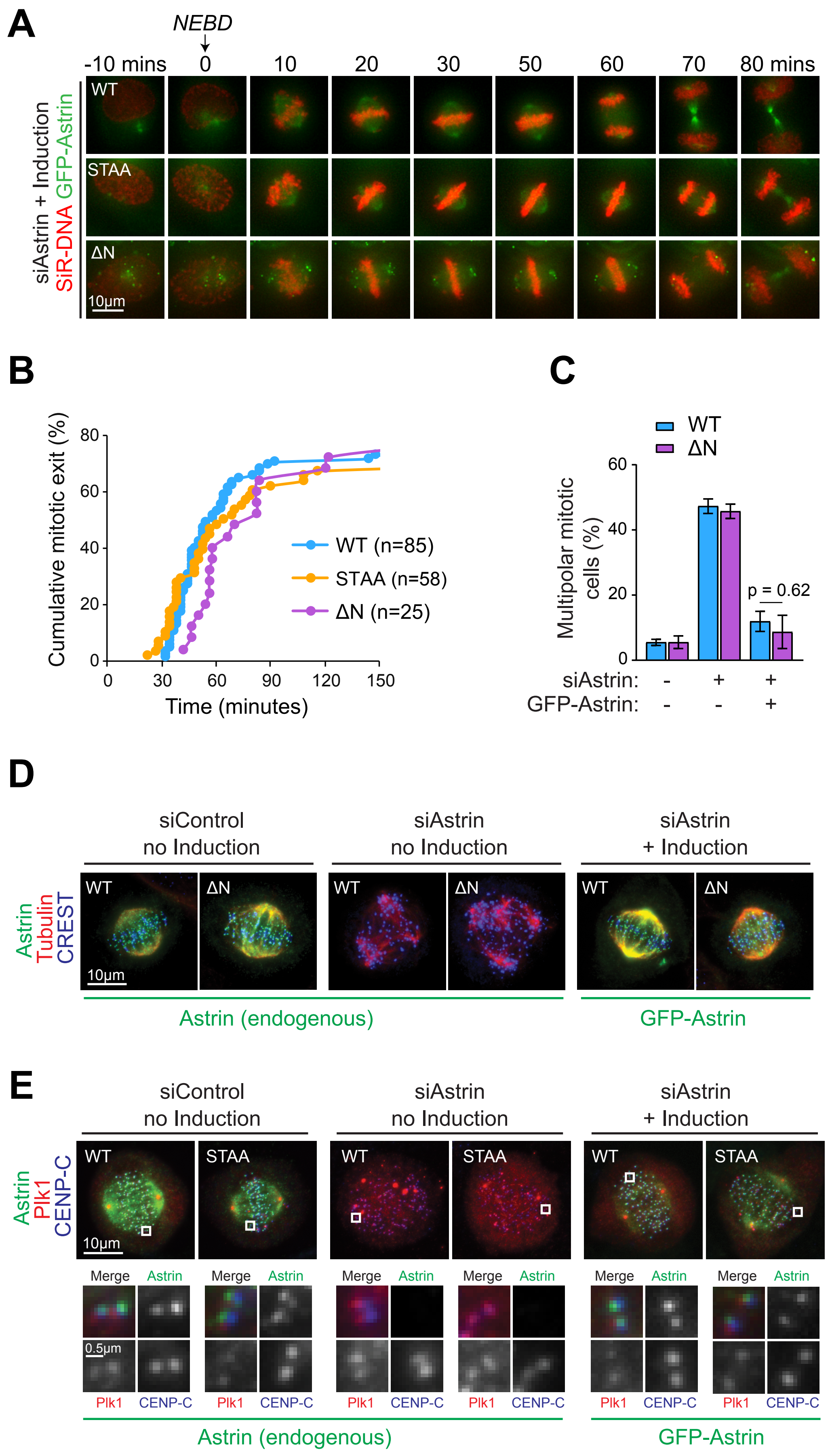

Journal of Cell Science • Supplementary information

Figure S3. The N-terminus of Astrin is not required for promoting spindle bipolarity. **A)** HeLa Flp-In TRex cells depleted of endogenous Astrin and induced to express GFP-Astrin^{WT}, GFP-Astrin^{STAA} or GFP-Astrin$^{\Delta N}$ (aa465-1193) were analysed by live cell imaging. Images were captured every 2 minutes. **B)** Analysis of the cells imaged in A). For each cell imaged, the time from nuclear envelope breakdown (NEBD) to anaphase onset was calculated. Each dot represents an individual cell; cells are from 4 (WT) or 2 (STAA, ΔN) independent experiments. **C)** HeLa Flp-In TRex cells were depleted of endogenous Astrin and induced to express GFP-Astrin^{WT} or GFP-Astrin$^{\Delta N}$, and stained for tubulin. The percentage of multipolar mitotic cells was quantified. Bars represent the mean ±SEM of 3 independent experiments, with 50-150 cells counted per condition per repeat. P-value was calculated by two-tailed Student t test. **D)** Representative images of the cells quantitated in C). **E)** In HeLa Flp-In TRex cells depleted of endogenous Astrin and induced to express GFP-Astrin^{WT} or GFP-Astrin^{STAA}, Plk1 kinetochore localization was visualised by immunofluorescence analysis.

Journal of Cell Science • Supplementary information

Figure S4

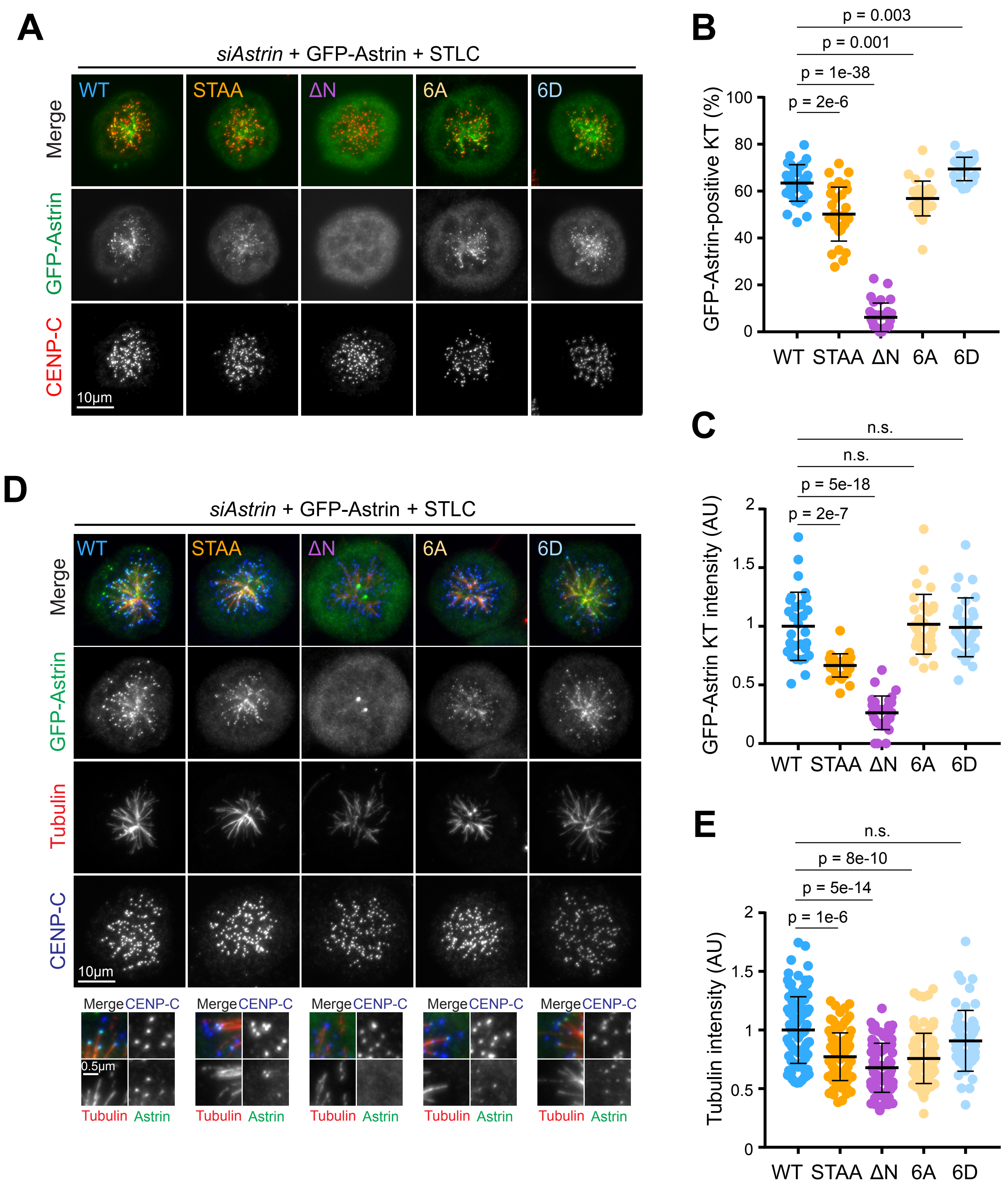

Journal of Cell Science • Supplementary information

Figure S4. A) HeLa Flp-In TRex cells depleted of endogenous Astrin and induced to express GFP-Astrin^{WT}, GFP-Astrin^{STAA}, GFP-Astrin$^{\Delta N}$, GFP-Astrin^{6A}, or GFP-Astrin^{6D} were arrested overnight with STLC. Cells were subjected to 9 min cold treatment immediately prior to fixation and then stained for Astrin, tubulin and CENP-C **B)** Quantitation of Astrin-positive kinetochores in A). The number of Astrin-positive kinetochores was counted and calculated as a percentage of visible kinetochores from CENP-C staining. For each condition 30 cells were analysed from 2 or 3 independent repeats. Each dot represents a single cell; error bars show mean ±SD. **C)** The intensity of GFP-Astrin-positive kinetochores was measured for the cells analysed in B). 20 Astrin-positive kinetochores were measured per cell. Each dot represents the mean kinetochore intensity of an individual cell; error bars show Mean ±SD. **D)** The total cold-stable tubulin intensity was measured in the cells analysed in B) and C).

Journal of Cell Science • Supplementary information
