# Supplementary material for: The association of Plk1 with the astrin–kinastrin complex promotes formation and maintenance of a metaphase plate
Source: J Cell Sci. 2021 Jan 8;134(1):jcs251025. doi: 10.1242/jcs.251025 (PMC7803464; doi:10.1242/jcs.251025)
Supplement: Reviewer comments [file joces-134-251025_review_history.pdf]

The association of Plk1 with the astrin-kinastrin complex promotes formation and maintenance of a metaphase plate

Zoë Geraghty, Christina Barnard, Pelin Uluocak and Ulrike Gruneberg
DOI: 10.1242/jcs.251025

Editor: David Glover

Review timeline

Original submission:	6 July 2020
Editorial decision:	5 August 2020
First revision received:	2 November 2020
Accepted:	30 November 2020

Original submission

<u>First decision letter</u>

MS ID#: JOCES/2020/251025

MS TITLE: The association of Plk1 with the Astrin-Kinastrin complex promotes formation and maintenance of a metaphase plate

AUTHORS: Zoe Geraghty, Christina Barnard, Pelin Uluocak, and Ulrike Gruneberg
ARTICLE TYPE: Short Report

We have now reached a decision on the above manuscript.

To see the reviewers' reports and a copy of this decision letter, please go to: https://submit-jcs.biologists.org and click on the 'Manuscripts with Decisions' queue in the Author Area. (Corresponding author only has access to reviews.)

As you will see, the reviewers raise a number of substantial criticisms that prevent me from accepting the paper at this stage. They suggest, however, that a revised version might prove acceptable, if you can address their concerns. If you think that you can deal satisfactorily with the criticisms on revision, I would be pleased to see a revised manuscript. We would then return it to the reviewers.

We are aware that you may currently be unable to access the lab to undertake experimental revisions. If it would be helpful, we encourage you to contact us to discuss your revision in greater detail. Please send us a point-by-point response indicating where you are able to address concerns raised (either experimentally or by changes to the text) and where you will not be able to do so within the normal timeframe of a revision. We will then provide further guidance. Please also note that we are happy to extend revision timeframes as necessary.

Please ensure that you clearly highlight all changes made in the revised manuscript. Please avoid using 'Tracked changes' in Word files as these are lost in PDF conversion.

I should be grateful if you would also provide a point-by-point response detailing how you have dealt with the points raised by the reviewers in the 'Response to Reviewers' box. Please attend to all of the reviewers' comments. If you do not agree with any of their criticisms or suggestions please explain clearly why this is so.

© 2020. Published by The Company of Biologists under the terms of the Creative Commons Attribution License (https://creativecommons.org/licenses/by/4.0/).

Reviewer 1

Advance summary and potential significance to field

This paper focuses on the regulation of the kinetochore and spindle-localized Astrin-Kinastrin complex by Plk1 kinase. Prior work from the Gruneberg lab and others has identified this complex and implicated it in diverse aspects of spindle formation and chromosome segregation. However, the mechanisms that regulate this complex, its localization, and its function remain unclear. This paper clearly and compellingly demonstrates that the Astrin-Kinastrin complex binds to Plk1 and is directly phosphorylated at residues throughout the protein. This paper presents a compelling combination of biochemistry, cell biology of Astrin localization (and phospho-specific antibodies against the identified sites), and a mutational analysis to test the contributions of these phosphorylation events. In particularly, the authors carefully highlight the role of this phosphorylation in promoting Astrin localization and stablizing the kinetochore-microtubule interface. I really enjoyed reading this paper, which I feel is carefully conducted and presented. I particularly liked the assay in Figure 4D/E, which highlights the importance of phosphorylation, as well as provides an exciting new assay for conceptualizing the role of this complex. It is rare that I prepare a review that doesn't identify flaws in a paper or critical additional experiments that are needed, but in this case I find myself highly enthusiastic about the paper with no major shortcomings. The take home message is important and useful for understanding the function and regulation of this outer kinetochore complex, the experiments are carefully conducted, and the paper is appropriately worded - highlighting the critical results without overstating things.
I strongly endorse publication of this paper and congratulate the authors on the excellent work.

Comments for the author

I only have some very minor suggestions:
• Figure 1F - it would be helpful to indicate which protein is FLAG tagged in the figure itself
• For Figure 2C, it would be helpful to highlight which residues are phosphorylated.

Reviewer 2

Advance summary and potential significance to field

In this manuscript by Geraghty et al. the authors extend on previous work from the same lab which identified an interaction between PLK1 and the Astrin-kinastrin complex (Dunsch et al.). Here, they follow-up on the interaction with PLK1 in particular and identify a classical CDK1-primed PLK1 binding site in the N-terminus of Astrin. PLK1 binding to Astrin does not appear have an effect on the formation of the Astrin-Kinastrin-DYNLL1 complex, nor does it result in multipolar spindle formation as is seen upon Astrin complex depletion. They went on to use phosphoproteomics to identify sites in the N-terminus of Astrin that are responsive to PLK1 inhibition and mutation of these sites collectively, or deletion of the entire N-terminus of Astrin reduced Astrin levels at the kinetochore but did not totally eliminate it. The authors finally suggest that the PLK-Astrin interaction is important for stability of KT-MT interactions and maintenance of metaphase arrests under conditions where the cells are prevented from exiting.
Overall, this manuscript offer a nice thorough description of the interaction between PLK1 and Astrin with identification of the binding site and demonstration of a direct binding between the PLK1 PBD and Astrin. The authors also show an effect of the N-terminal region (in of itself) in tethering Astrin to kinetochores. Nevertheless, the data linking the PLK1 interaction to the phosphorylation sites and hence to the reduction in Astrin kinetchore levels are somewhat weak and do not always fit together somewhat dampening my enthusiasm for this manuscript.

Comments for the author

Major comments:
1. Effect of PLK1 inhibition/depletion versus the inhibition of PLK1 binding to Astrin: Plk1 inhibition appears to completely abolish Astrin kinetochore localization (Fig. 1b) whereas inhibiting the PLK1-Astrin interaction only reduces Astrin binding by a very small but statistically significant amount at least insome experiments (Fig.3G versus 4C). This suggests that PLk1 influences Astrin

© 2020. Published by The Company of Biologists under the terms of the Creative Commons Attribution License (https://creativecommons.org/licenses/by/4.0/).

localization indirectly in a more significant manner than through direct binding, and that the authors may be overextimating the role of this interaction of Astrin. At the very least the authors should discsuss this difference and offer alternative explantations for their observatinos. What else could PLK1 be doing that is promoting Astrin localization

2. Related to the above statement: the authors provide no direct evidence that the sites identified are indeed PLK1 sites. The observation that they are lost upon PLk1 inhibition does not immediately qualify them as plk1 sites. Indeed previous work has clearly shown that PLK1 inhibition can indirectly modulate the activity of other kinases such as Aurora family kinases (Santamaria et al 2011 and Kettenbach 2011, 2018). Moreover, the statement that the sites identified adhere loosely to the plk1 consensus (line 153), is very generous, unless I am missing something completely here with the exception of S353. S411 looks like a Aurora site for example and neither 157 not 159 look like the consensus. I suggest that the authors demonstrate direct phosphorylation of these site in in vitro reactions. This should be readily doable considering all the reagents they have at their disposal, including their phosphoantibodies.

3. In figure 4 where the role of plk1-binding deficient astrin on Kinetochore-microtubule interactions are being explored, again the authors provide only indirct evidence. They demonstrate in Fig. 4A-C that less of the PLk-binding mutant of astrin associated with cold stable K-fibres (and not the other way round as they are claiming/concluding and as the subtitle of this section suggests. In addition, the MG13 experiment is also indirect. The authors should consider measuring K-fibre stability directly in their stable inducible cells lines, if they want to claim an effect of Astrin on kinetochore-microtubule interaction stability.

Minor comments:

1. In Line 194: Authors should refer to both Fig 3G and Fig 2F
2. In Fig. 3D, and line 187, the authors should refer to the exact phospohsite/phosphoantibody they mean.
3. Normalization of Astrin levels. Is is not clear why the authors chose to measure total Astrin levels, and did not normalize to a kinetochore marker. The methods section describes how this was done but not why.
4. Line 367: should say mean not amean.

<u>Reviewer 3</u>

Advance summary and potential significance to field

This manuscript extends previous work from this group on the kinetochore-associated protein, astringent. Having previously shown that Plk1 is present in a complex with astrin and kinastrin, the authors here identify a binding site in astrin (S110/T111) that allows direct interaction with Plk1 and show that this interaction is required to generate stable kinetochore-microtubule attachments and prolonged metaphase plate alignment. They also identify a number of Plk1 phosphorylation sites in astrin although their functional significance in terms of kinetochore-microtubule attachment is not examined.

Comments for the author

As it stands, this study is well executed and adds valuable information to understanding how Plk1 promotes error-free cell division particularly during prolonged mitosis. Of course, it would be very nice to show the contribution of the Plk1 phosphosites in astrin to its mitotic function, particularly as the authors have already generated a phosphonull mutant. However, I appreciate that in the current circumstances it may be difficult to undertake these additional experiments. Therefore, if the authors can respond positively to the comments below, I would be happy to recommend publication in the Journal of Cell Science.

Abstract. As the authors have not shown the functional role of the Plk1 phosphorylation sites in astrin, I suggest removing the final sentence proposing that astrin phosphorylation by Plk1 contributes to effective microtubule-kinetochore attachment.

Figure 1. Panel B suggests that upon depletion of Plk1, astrin becomes strongly associated with spindle microtubules. Do the authors have an explanation for this? While panel D demonstrates that

© 2020. Published by The Company of Biologists under the terms of the Creative Commons Attribution License (https://creativecommons.org/licenses/by/4.0/).

mutations in the Plk1 PBD interfere with binding to astrin, it does not indicate that only phosphorylated astrin binds to Plk1 as stated in the text (line 108). This is an assumption based on the fact that PBDs have been shown to bind phosphorylated sequences; this should be made clear in the text. The Far Western blots shown in Panel G should include astrin proteins that have not been incubated with Cdk1, or Cdk1 together with a Cdk1 inhibitor. This is an important control to show that binding in this context is dependent on Cdk1 phosphorylation.

Figure 2. The phosphospecific astrin antibody gives staining patterns that are very similar to the total astrin antibody in response to Plk1 inhibition, astrin depletion and across the different stages of mitosis. Hence, I am concerned that this antibody may not be selective for the phosphorylated version of Astrin. In panel F, what is the significance of the difference in staining between astrin and phospho-astrin upon Plk1 inhibition? Does this antibody work in Western blotting where it can be used to demonstrate reactivity for Astrin only after incubation with Plk1 in vitro? The latter data would provide significant reassurance of the selectivity of this antibody.

Figure 3. In the earlier Figure 1B, it was shown that depletion of Plk1 led to complete loss of astrin from kinetochores suggesting that this localization was entirely dependent on Plk1. However, the Astrin-STAA mutant that cannot bind Plk1 only exhibited a modest decrease in localization to kinetochores (Fig. 3G). How do the authors explain this apparent discrepancy?

Figure 4. The authors describe having generated a GFP-Astrin-6A mutant that has the four potential Plk1 phosphorylation sites mutated as well as mutations in the two sites adjacent to S411. The fact that this mutant showed a similar reduction in kinetochore localization as the PBD mutant is a valuable result and potentially warrants moving this data from the supplementary to main results. However, the key accompanying data would be whether this mutant also impairs microtubule-kinetochore attachments and maintenance of a metaphase plate. The manuscript would be significantly enhanced if the outcome of this experiment could be added as it would point to a role for Plk1 phosphorylation of astrin.

Additional points:
- Lines 73-75. The sentence beginning “This role is promoted by Astrin delivering …” should be revised as its meaning is currently unclear.
- Lines 154-156 state that the S411 phosphorylation site could not be assigned unambiguously due to the presence of T412 and S413. So why then do the Results seem to state clearly that the site is S411?
- Line 605 is missing the word ‘of’.

First revision

Author response to reviewers' comments

SIR WILLIAM DUNN SCHOOL OF PATHOLOGY

Oxford, 30th October 2020

The Journal of Cell Science
To the Editor,
We would like to thank the reviewers very much for their constructive comments and suggestions on our manuscript **‘The association of Plk1 with the Astrin-Kinastrin complex promotes formation and maintenance of a metaphase plate’.** We have now revised the manuscript extensively to address the points that were raised, including the addition of several new pieces of data. In brief, we have added

- immunofluorescence data confirming that kinetochores in Plk1 depleted cells are

© 2020. Published by The Company of Biologists under the terms of the Creative Commons Attribution License (https://creativecommons.org/licenses/by/4.0/).

unattached
- an updated Far Western experiment with extra controls
- *in vitro* kinase assay data showing that Astrin is a Plk1 substrate
- immunofluorescence data supporting that *in vivo* Astrin phospho-sites pS157 and pS159 are directly phosphorylated by Plk1 and not other mitotic kinases
- live cell imaging data of Astrin phospho-null and phospho-mimetic mutants
- an analysis of microtubule cold-stability as a measure of K-fibre stability in cells expressing different variants of Astrin.

We feel that the manuscript has been significantly improved by the text revisions and additional new data, and we hope very much that you will now find our revised manuscript suitable for publication in The Journal of Cell Science.
Please find a detailed reply to all the issues that the reviewers raised below.

Reviewer 1
I strongly endorse publication of this paper and congratulate the authors on the excellent work.
We thank the referee for the positive evaluation of our manuscript.
Reviewer 1 Comments for the Author:
I only have some very minor suggestions:
- Figure 1F - it would be helpful to indicate which protein is FLAG tagged in the figure itself
- For Figure 2C, it would be helpful to highlight which residues are phosphorylated.

We have made the requested changes.

Reviewer 2
Major comments:
1. Effect of PLK1 inhibition/depletion versus the inhibition of PLK1 binding to Astrin: Plk1 inhibition appears to completely abolish Astrin kinetochore localization (Fig. 1b) whereas inhibiting the PLK1-Astrin interaction only reduces Astrin binding by a very small but statistically significant amount at least insome experiments (Fig.3G versus 4C). This suggests that PLk1 influences Astrin localization indirectly in a more significant manner than through direct binding, and that the authors may be overextimating the role of this interaction of Astrin. At the very least, the authors should discsuss this difference and offer alternative explantations for their observatinos. What else could PLK1 be doing that is promoting Astrin localization

Plk1 is known to be a major factor promoting microtubule-kinetochore attachment formation, however, the molecular targets have not all been identified. Since Astrin localises exclusively to attached kinetochores, there is an indirect effect of Plk1 depletion on Astrin localisation because microtubule-kinetochore attachments are not formed when cells enter mitosis in the absence of Plk1. We had explained this in the original manuscript but have now stressed this even more in the revised version of the manuscript. The revised text now reads: “In contrast, Astrin was absent from kinetochores in Plk1 depleted cells (Figure 1B) which lack the stable kinetochore attachments required for Astrin kinetochore localisation (Lenart et al., 2007; Schmidt et al., 2010) as evidenced by high levels of the spindle checkpoint protein MAD1 (Figure S1B).”
To emphasise that Plk1-depleted cells arrest in mitosis with unattached kinetochores, thus precluding Astrin localisation, we have included extra data confirming that these kinetochores have active Spindle Assembly Checkpoint signalling as visualised by the SAC protein Mad1 (new Figure S1B).
2. Related to the above statement: the authors provide no direct evidence that the sites identified are indeed PLK1 sites. The observation that they are lost upon PLk1 inhibition does not immediately qualify them as plk1 sites. Indeed previous work has clearly shown that PLK1 inhibition can indirectly modulate the activity of other kinases such as Aurora family kinases (Santamaria et al 2011 and Kettenbach 2011, 2018). Moreover, the statement that the sites identified adhere loosely to the plk1 consensus (line 153), is very generous, unless I am missing something completely here, with the exception of S353. S411 looks like a Aurora site for example and neither 157 not 159 look like the consensus. I suggest that the authors demonstrate direct phosphorylation of these site in in vitro reactions. This should be readily doable considering all the reagents they have at their disposal, including their phosphoantibodies.

We agree that our discussion of the Plk1 sites on Astrin was rather too concise. As the referee states, S353 in Astrin fits quite well with the Plk1 consensus motif. The S157/S159 peptide is

© 2020. Published by The Company of Biologists under the terms of the Creative Commons Attribution License (https://creativecommons.org/licenses/by/4.0/).

interesting because phosphorylation of S157 by Plk1 would turn this residue into an acidic one, thus creating a Plk1 consensus motif for S159. For S411 our mass spec analysis was not able to unequivocally determine whether the phosphorylated residue is on S411, T412 or S413. While S411 would indeed fit the requirements of an Aurora site, the same is not true for T412 and S413. We have now made it clearer in the revised manuscript that the assignment of the phosphorylated residue in this peptide is not unambiguous. The revised manuscript now also contains an extended discussion of the sequences of the PLk1 sites on Astrin:

"Interestingly, in the peptide containing S157 and S159, phosphorylation of S157 by Plk1 introduces a negative charge at the -2 position to S159 and therefore creates a sequence closer to the canonical Plk1 consensus motif for phosphorylation of S159. S411 could not be assigned with absolute certainty as the phosphorylated residue because of the presence of two S/T residues following S411 (Figure S2A)."

As for the experimental evidence, our analysis of cells depleted of endogenous Astrin and expressing Plk1-binding site deficient Astrin in Figure 3 of the original manuscript clearly demonstrated that loss of Plk1 binding to Astrin resulted in loss of staining with our pS157/159 phospho-specific antibody. Because our experiment only removes Astrin-bound Plk1 but leaves all other Plk1 intact and active, the most parsimonious explanation of this result is that Plk1 is the kinase that phosphorylates these residues. Nevertheless, to experimentally test more rigorously whether another mitotic kinase could be directly responsible for the phosphorylation of the pS157/159 residues that we had identified on Astrin, we have now added an additional figure panel in Figure S2B in which we analysed the staining patterns for the Astrin phospho-specific pS157/159 antibody in mitotic cells treated with inhibitors of Plk1, MPS1, Aurora B or Aurora A. Our analysis showed that while Plk1 inhibition abolished the phospho-specific staining at kinetochores, inhibition of MPS1, Aurora B or Aurora A had no or only minor effects on phospho-Astrin staining (new Figure panels S2B-C). This result further strengthens the conclusion that Plk1 is responsible for directly phosphorylating these residues. Additionally, as suggested by the reviewer we have included in vitro kinase assays (new Figure 2C) to provide further evidence that Plk1 directly phosphorylates the N-terminus of Astrin.

3. In figure 4 where the role of plk1-binding deficient astrin on Kinetochore-microtubule interactions are being explored, again the authors provide only indirct evidence. They demonstrate in Fig. 4A-C that less of the PLk-binding mutant of astrin associated with cold stable K-fibres (and not the other way round as they are claiming/concluding and as the subtitle of this section suggests. In addition, the MG13 experiment is also indirect. The authors should consider measuring K-fibre stability directly in their stable inducible cells lines, if they want to claim an effect of Astrin on kinetochore-microtubule interaction stability.

In order to assess K-fibre stability more directly as requested by the referee, we have now measured the total tubulin intensity of the cold-stable microtubules present in HeLa cells depleted of endogenous Astrin and expressing the different Astrin mutants. In this cold assay, the bulk of spindle microtubules are depolymerised, while microtubules associated with kinetochores (kinetochore fibres) are selectively protected from depolymerisation (Rieder, 1981), thus providing a snapshot of stable kinetochore-microtubule attachments. Our results confirm that in mitotic cells expressing Plk1-binding deficient Astrin or Astrin lacking the N-terminal domain, or lacking the Plk1 phosphorylation sites, less cold-stable tubulin is present than in cells with WT Astrin or Plk1 site phospho-mimetic Astrin. This trend mirrors the apparent number of attachments as quantified by the percentage of kinetochores with Astrin present (Figure S4B) confirming that Astrin-positive kinetochores are indeed associated with microtubules. Taken together, these data are consistent with our model that Plk1-binding mutant Astrin or non-phosphorylatable Astrin is defective in the normal maintenance of these kinetochore-microtubule associations, resulting in fewer attached kinetochores in STLC arrested cells. These results are also in keeping with the results of our functional assays measuring by live cell imaging the maintenance of a metaphase plate in these cells, which further support the idea that kinetochore-microtubule attachments formed without Plk1 phosphorylation of the Astrin N-terminal domain are more susceptible to dissociation.

Minor comments:

1. In Line 194: Authors should refer to both Fig 3G and Fig 2F

This has been changed.

2. In Fig. 3D, and line 187, the authors should refer to the exact phospohsite/phosphoantibody they mean.

This has been fixed.

3. Normalization of Astrin levels. Is is not clear why the authors chose to measure total Astrin levels, and did not normalize to a kinetochore marker. The methods section describes how this was

© 2020. Published by The Company of Biologists under the terms of the Creative Commons Attribution License (https://creativecommons.org/licenses/by/4.0/).

done but not why.
We found that varying levels of total GFP-Astrin expression affected the levels of GFP-Astrin localised to kinetochores. To exclude that different levels of GFP-Astrin at kinetochores were caused by differences in the GFP-Astrin expression levels (rather than the properties of the mutant protein), only cells with equivalent levels of GFP-Astrin expression were analysed.
This had been explained (albeit very briefly) in the original Material and Methods section but has now been expanded to say:
“To ensure that the analysis of GFP-Astrin expressing HeLa Flp-in cells cells was not affected by varying GFP-Astrin expression levels potentially leading to different GFP-Astrin kinetochore levels, these cells were fixed in PFA to preserve the cytoplasmic pool of Astrin, the intensities of the whole cells were measured, and only cells with equivalent expression levels were included in the analysis.”
4. Line 367: should say mean not amean.
This has been fixed.

Reviewer 3
As it stands, this study is well executed and adds valuable information to understanding how Plk1 promotes error-free cell division particularly during prolonged mitosis. Of course, it would be very nice to show the contribution of the Plk1 phosphosites in astrin to its mitotic function, particularly as the authors have already generated a phosphonull mutant. However, I appreciate that in the current circumstances it may be difficult to undertake these additional experiments. Therefore, if the authors can respond positively to the comments below, I would be happy to recommend publication in the Journal of Cell Science.
We thank the referee for their positive evaluation of our work. We have now included additional live cell imaging experiments which show that cells expressing Astrin-phospho-null mutants display a similar defect in maintaining a metaphase plate for prolonged periods of time as cells expressing Plk1-binding deficient Astrin. These results confirm our idea that the phosphorylation of the N-terminus of Astrin by Plk1 contributes positively to stable microtubule-kinetochore attachments and therefore metaphase plate maintenance.
Abstract. As the authors have not shown the functional role of the Plk1 phosphorylation sites in astrin, I suggest removing the final sentence proposing that astrin phosphorylation by Plk1 contributes to effective microtubule-kinetochore attachment.
Since we have now provided more evidence to support this statement we have left the abstract as it is.
Figure 1. Panel B suggests that upon depletion of Plk1, astrin becomes strongly associated with spindle microtubules. Do the authors have an explanation for this?
We reproducibly obtained this result but currently have no definite explanation for this. We speculate that in the absence of stable microtubule-kinetochore attachments (which require Plk1) Astrin localises more strongly to microtubules because it cannot go to kinetochores.
While panel D demonstrates that mutations in the Plk1 PBD interfere with binding to astrin, it does not indicate that only phosphorylated astrin binds to Plk1 as stated in the text (line 108). This is an assumption based on the fact that PBDs have been shown to bind phosphorylated sequences; this should be made clear in the text.
Our Western blot shows that only phosphorylated Astrin is pulled down by the PBD, see lane 5 in panel D blotted for Astrin: only the top Astrin band is precipitated by WT PBD. We have revised the text to explain this more clearly. The text now reads: “GFP pulldowns from cells transiently expressing GFP-Plk1^{WT} or GFP-Plk1^{HKAA}, lacking two PBD residues required for phospho-specific binding, (Elia et al., 2003b; Hanisch et al., 2006), confirmed that the interaction between Plk1 and Astrin depended on an intact PBD, and that only the phosphorylated, high molecular weight form, of Astrin interacted with Plk1 (Figure 1D, arrow).” We have also included an arrow in the figure to point out the phosphorylated, high molecular weight form of Astrin that is pulled down by Plk1.
The Far Western blots shown in Panel G should include astrin proteins that have not been incubated with Cdk1, or Cdk1 together with a Cdk1 inhibitor. This is an important control to show that binding in this context is dependent on Cdk1 phosphorylation.
We have replaced the Far Western blot in Figure 1G with an updated version which contains the requested controls.
Figure 2. The phosphospecific astrin antibody gives staining patterns that are very similar to the total astrin antibody in response to Plk1 inhibition, astrin depletion and across the different stages of mitosis. Hence, I am concerned that this antibody may not be selective for the phosphorylated version of Astrin. In panel F, what is the significance of the difference in staining between astrin

© 2020. Published by The Company of Biologists under the terms of the Creative Commons Attribution License (https://creativecommons.org/licenses/by/4.0/).

and phospho-astrin upon Plk1 inhibition? Does this antibody work in Western blotting where it can be used to demonstrate reactivity for Astrin only after incubation with Plk1 in vitro? The latter data would provide significant reassurance of the selectivity of this antibody.

We agree with the referee that the specificity of phospho-specific antibodies has to be very carefully controlled. We had already shown in the original manuscript that our phospho- specific antibody only produces a staining in the presence of Astrin protein and Plk1 activity. Importantly, phospho-staining is entirely lost from kinetochores in the absence of Plk1 activity despite the fact that Astrin is still clearly present, albeit at reduced levels (Figure 2D, 2G).We have now added a new figure panel showing that our phospho-specific antibody does not recognise a version of Astrin in which the Plk1 phosphorylation sites have been mutated to alanine (new Figure panel S2E). We have also included an additional experiment which compares the Astrin phospho-specific staining in cells that have been treated with inhibitors of the mitotic kinases MPS1, Plk1, Aurora B and Aurora A. In this situation, only Plk1 inhibition resulted in the loss of the phospho-specific staining. Taken together, we are confident that our antibody only recognises phosphorylated Astrin. Unfortunately, the antibody does not work well on Western blots, so we were not able to carry out the in vitro experiments suggested by the reviewer.

Figure 3. In the earlier Figure 1B, it was shown that depletion of Plk1 led to complete loss of astrin from kinetochores suggesting that this localization was entirely dependent on Plk1. However, the Astrin-STAA mutant that cannot bind Plk1 only exhibited a modest decrease in localization to kinetochores (Fig. 3G). How do the authors explain this apparent discrepancy?

Plk1 is known to be a major factor promoting microtubule-kinetochore attachment formation, however, the molecular targets have not all been identified. Since Astrin localises preferentially to attached kinetochores, there is a significant indirect effect of Plk1 inhibition or Plk1 depletion on Astrin localisation because microtubule-kinetochore attachments are not formed. The replacement of endogenous Astrin with Plk1-binding deficient Astrin only removes the direct effects of Plk1 on Astrin and therefore has a smaller effect on Astrin biology.

Figure 4. The authors describe having generated a GFP-Astrin-6A mutant that has the four potential Plk1 phosphorylation sites mutated as well as mutations in the two sites adjacent to S411. The fact that this mutant showed a similar reduction in kinetochore localization as the PBD mutant is a valuable result and potentially warrants moving this data from the supplementary to main results. However, the key accompanying data would be whether this mutant also impairs microtubule-kinetochore attachments and maintenance of a metaphase plate. The manuscript would be significantly enhanced if the outcome of this experiment could be added as it would point to a role for Plk1 phosphorylation of astrin.

We have now included additional live cell imaging experiments which show that cells expressing Astrin-phospho-null mutants display a similar defect in maintaining a metaphase plate for prolonged periods of time as cells expressing Plk1-binding deficient Astrin (new Figure panels 4D and 4E). These results confirm our idea that the phosphorylation of the N- terminus of Astrin by Plk1 contributes positively to microtubule-kinetochore attachment maintenance and hence metaphase plate preservation.

Additional points:

- Lines 73-75. The sentence beginning “This role is promoted by Astrin delivering …” should be revised as its meaning is currently unclear.

This sentence has been reworded to “This process is aided by a specific pool of the PP1 phosphatase which is delivered by Astrin to kinetochores (Conti et al., 2019).”

- Lines 154-156 state that the S411 phosphorylation site could not be assigned unambiguously due to the presence of T412 and S413. So why then do the Results seem to state clearly that the site is S411?

The text now refers to this site as S411/T412/S413.

- Line 605 is missing the word ‘of’.

This has been fixed.

Thank you again for considering our manuscript. With best wishes,
Ulrike Gruneberg, PhD

© 2020. Published by The Company of Biologists under the terms of the Creative Commons Attribution License (https://creativecommons.org/licenses/by/4.0/).

Second decision letter

MS ID#: JOCES/2020/251025

MS TITLE: The association of Plk1 with the Astrin-Kinastrin complex promotes formation and maintenance of a metaphase plate

AUTHORS: Zoe Geraghty, Christina Barnard, Pelin Uluocak, and Ulrike Gruneberg
ARTICLE TYPE: Short Report

I am happy to tell you that your manuscript has been accepted for publication in Journal of Cell Science, pending standard ethics checks.

Reviewer 1

Advance summary and potential significance to field

This paper explores the regulation of a critical kinetochore complex - the Astrin/Kinastrin complex. The authors define a clear phosphorylation and control by Plk1 kinase that contributes to its roles in the maintenance of the metaphase plate.

Comments for the author

I continue to be enthusiastic and supportive of this paper. For this revised version, the authors have made multiple changes to the text and figures in response to the substantive comments from the other reviewers. In my opinion, these changes and additions address each of the concerns from the other reviewers and have further improved what is a solid paper. I recommend publication of this paper in JCS.

Reviewer 3

Advance summary and potential significance to field

The manuscript nicely describes convincing new evidence that an additional role for the mitotic kinase PLK1 is to stabilize microtubule-kinetochore attachments through recruitment and phosphorylation of the astrin protein complex.

Comments for the author

The authors have added valuable new data and responded appropriately to all the points suggested in my original review. I am therefore very happy to now recommend publication of this excellent study.

© 2020. Published by The Company of Biologists under the terms of the Creative Commons Attribution License (https://creativecommons.org/licenses/by/4.0/).
